# Supplementary material for: Zika virus replicates in adult human brain tissue and impairs synapses and memory in mice
Source: Nat Commun. 2019 Sep 5;10:3890. doi: 10.1038/s41467-019-11866-7 (PMC6728367; doi:10.1038/s41467-019-11866-7)
Supplement: Supplementary file 1 — Supplementary Information [file 41467_2019_11866_MOESM1_ESM.pdf]

Zika virus replicates in adult human brain tissue and impairs synapse function and memory in adult mice

Figueiredo, CP, Barros-Aragão, FGQ and Neris, RLS et al, Nature Communications, 2019

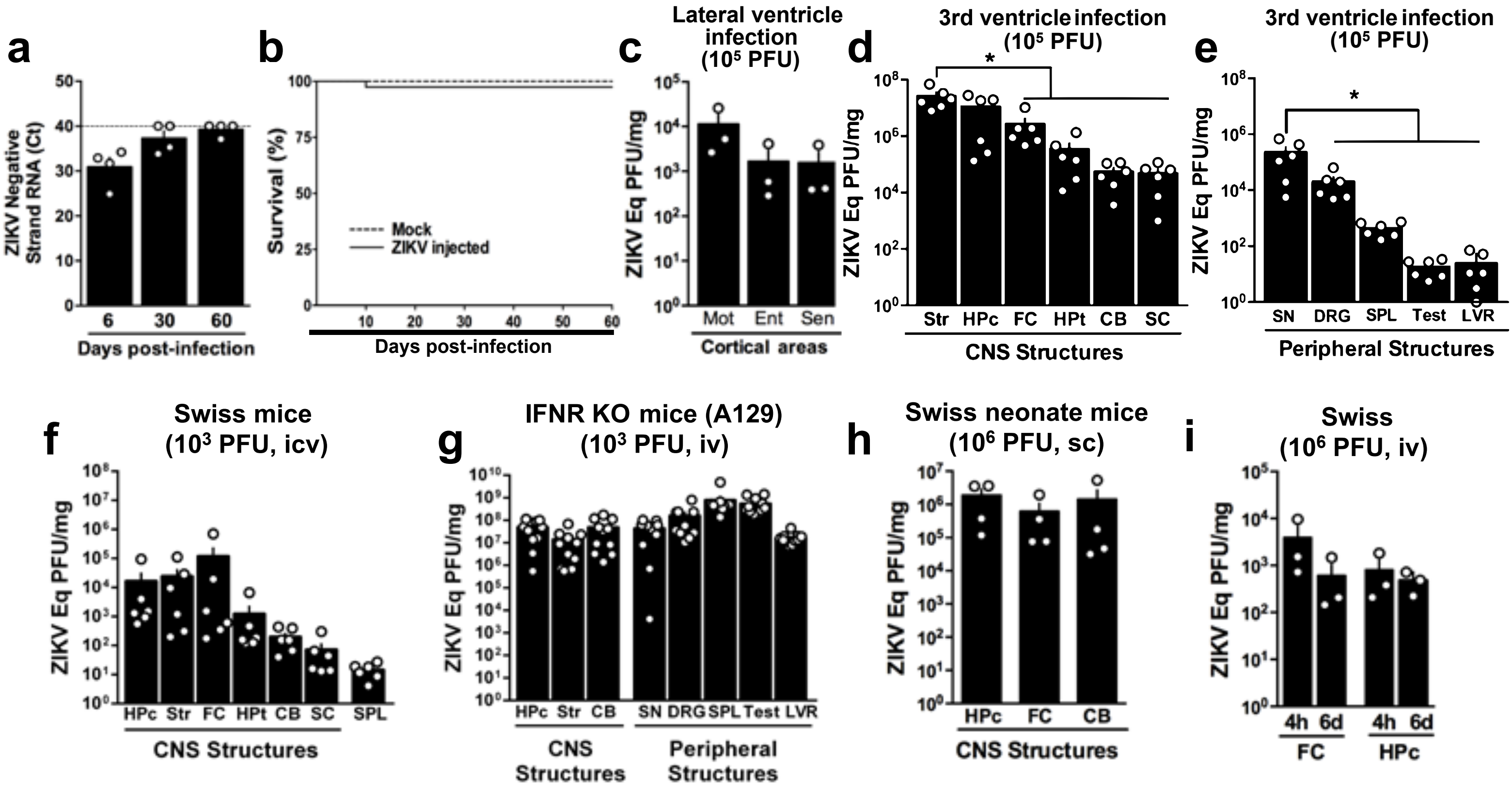

Supplementary Figure 1. ZIKV detection and viral distribution after infection in different animal models. (a-c) Adult Swiss mice received an i.c.v. infusion of 10^5 PFU ZIKV or mock medium. (a) At the indicated dpi, brains were processed for determination of ZIKV negative strand RNA by qPCR (N=4 mice/time-point). Ct values ≥ 40 (i.e., below the detection limit) were represented as Ct = 40 in the graph. (b) Survival rates were monitored up to 60dpi (N=20 mice/group; data represent one of two independent experiments). (c) ZIKV mRNA load in motor (Mot), entorhinal (Ent) and sensory (Sen) cortex at 6dpi (N=3 mice/group). (d-e) Distinct CNS (d) or peripheral (e) structures were isolated 6 days post infusion of ZIKV (10^5 PFU) into the third brain ventricle and processed for determination of viral load by qPCR (d: $F(5,30)=4.020$; *p=0.0031 Str vs FC; p=0.0011 Str vs HPt; p=0.0009 Str vs. CB; p=0.0009 Str vs SC; in e: $F(4,25)=5.261$; *p=0.0193 SN vs DRG; p=0.0095 SN vs SPL; p=0.0094 SN vs LVR; p=0.0094 SN vs Testes; one-way ANOVA followed by Bonferroni, N=6 mice/group). (f) Adult Swiss mice received an i.c.v. infusion of ZIKV (10^3 PFU) into the lateral ventricle. Distinct CNS structures and spleen were isolated at 6dpi and processed for determination of viral load by qPCR (N=6 mice/group). (g) Interferon Type I receptor knockout mice (A129) received ZIKV i.v. (10^3 PFU) and distinct CNS or peripheral structures were isolated and processed at 6dpi for determination of viral load (N=10 mice for SN and DRG, 11 other structures.) (h) Neonatal Swiss mice were infused s.c. with ZIKV (106 PFU) and brain structures were isolated at 12dpi for determination of viral load by qPCR (N=4 mice/group). (i) Adult Swiss mice received an i.v. infusion (retroorbital) of ZIKV (10^6 PFU), brain structures were isolated at 6dpi for determination of viral load by qPCR (N=3 mice/group). Bars represent means±SEM. Symbols represent individual mice. HPc (hippocampus); FC (frontal cortex); Str (striatum); CB (cerebellum); HPt (hypothalamus); SC (spinal cord); SPL (spleen); LVR (liver); DRG (dorsal root ganglion); SN (sciatic nerve); test (testes). Source data are provided as Source Data File.

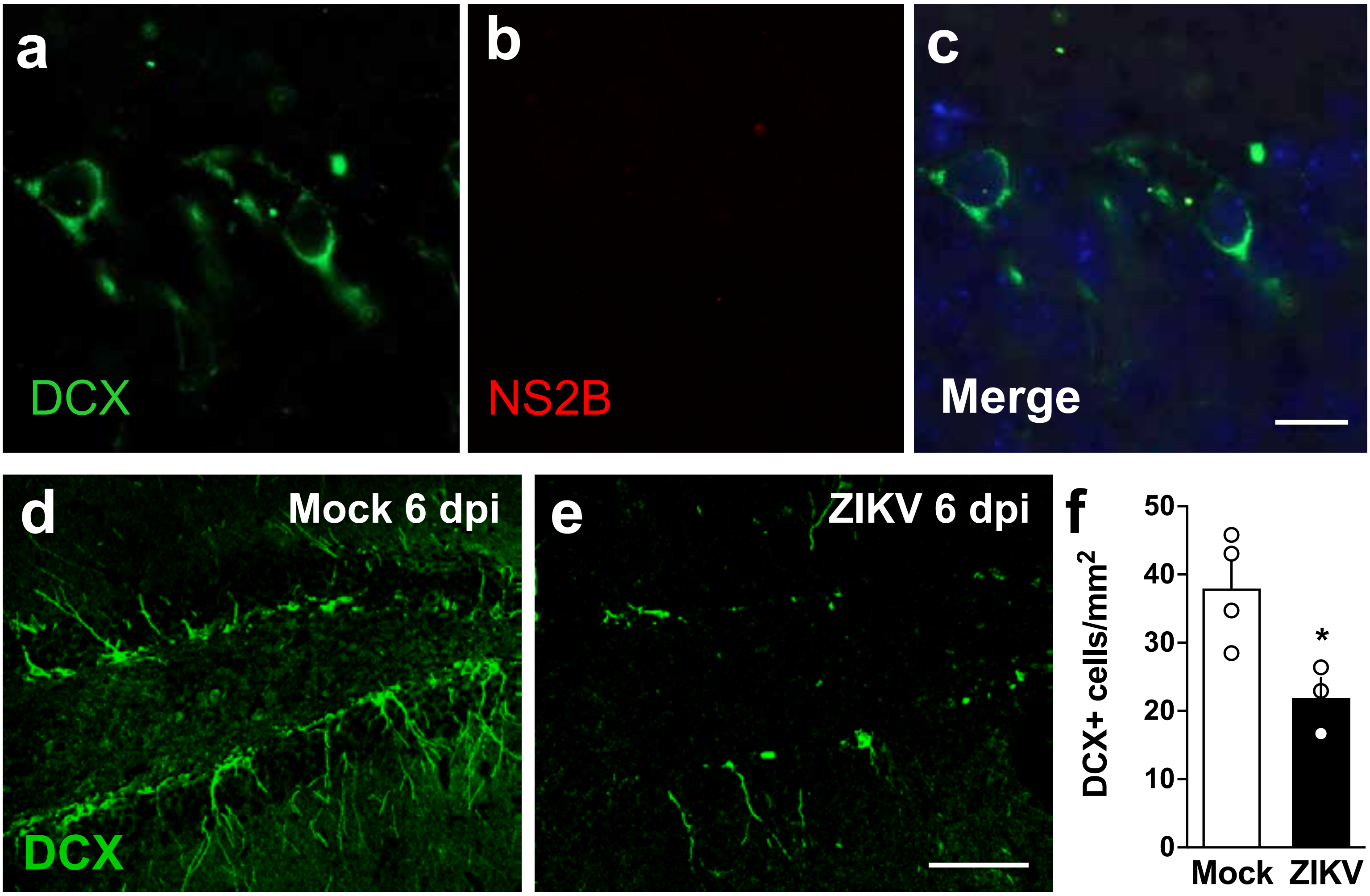

Supplementary Figure 2. Reduced numbers of neuronal progenitor cells in the dentate gyrus of ZIKV-infected mice. Adult Swiss mice received an i.c.v. infusion of 105 PFU ZIKV or mock medium. (a-c) Representative images of double immunolabeling for ZIKV (NS2B protein, red) and doublecortin (DCX, green) in hippocampus dentate gyrus at 6dpi. Scale bar=15 µm. (d, e) Representative images showing doublecortin (DCX) immunolabeling in the dentate gyrus (DG) of mock- or ZIKV-infused mice at 6dpi. Scale bar=50 µm. (f) Bars represent the number of DCX positive-cells in the dentate gyrus of mock- or ZIKV-infused mice at 6dpi ($t = 3.049$, $p = 0.0285$, Student's t-test). N=4 (mock) and 3 (ZIKV). Bars represent means ± SEM. Symbols represent individual mice. Source data from panel f is provided as Source Data File.

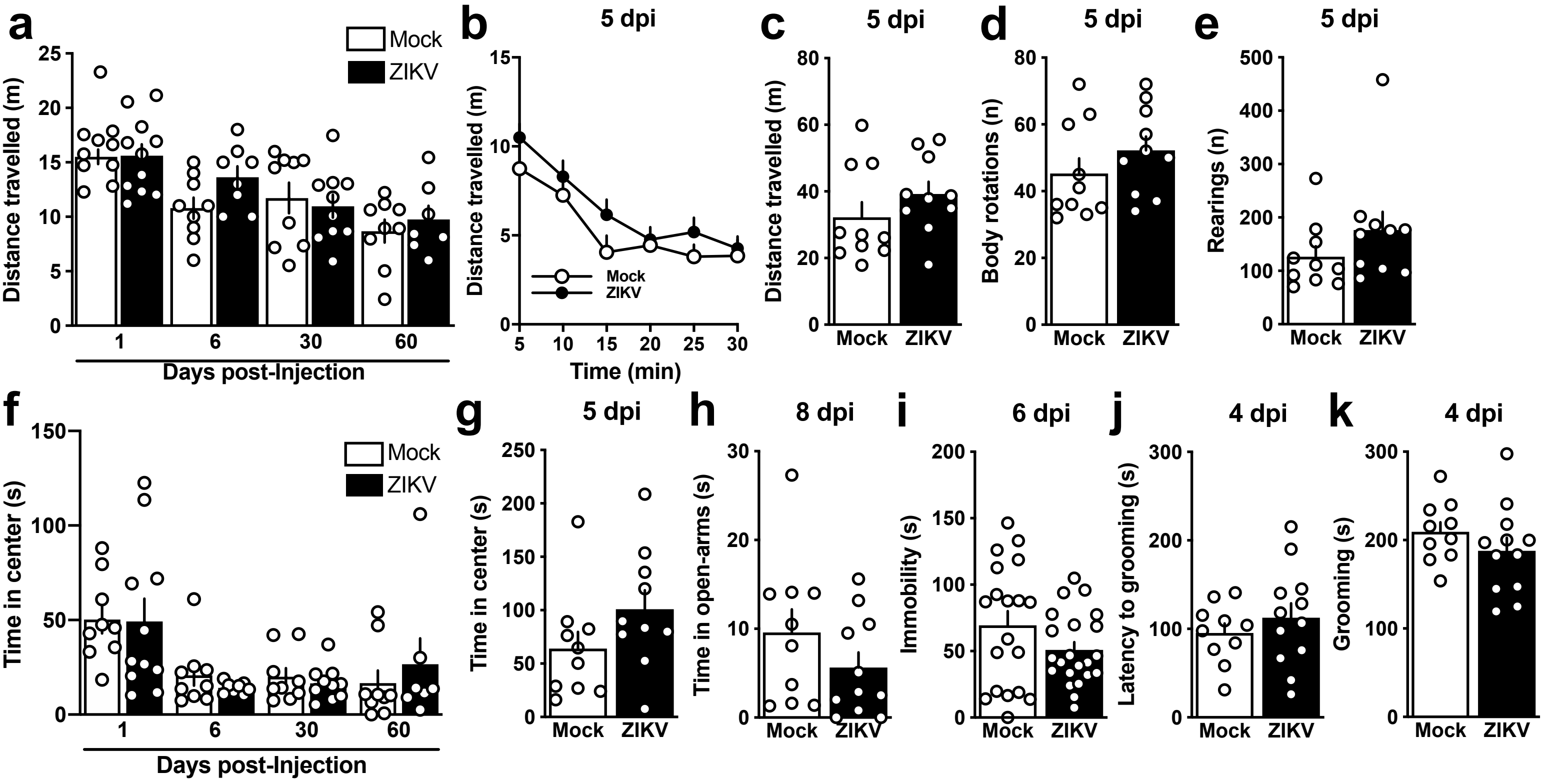

Supplementary Figure 3. ZIKV does not affect locomotor/exploratory activity in an open field, or induce anxiety- or depressive-like behaviors in adult mice. Adult Swiss mice received an i.c.v. infusion of 10^5 PFU ZIKV or mock medium. (a-g) Animals were tested in an open field arena at the indicated dpi. Total distance traveled (a), habituation to the open field during 30 min (b), total body rotations (d), number of rearings (e), and time spent at the center of the open field arena (f, g) were measured. (h-k) Animals were tested in the elevated plus maze, tail suspension and sucrose splash tests. Time spent in the open arms of the elevated plus maze (h), immobility time during 6 min of tail suspension (i), and latency to start (j) and total self-grooming behavior (k) after sucrose splashing. Bars represent means ± SEM. Symbols correspond to individual mice. In a and f: N = 9 (mock), 11 (1dpi), 8 (15dpi), 10 (30dpi) and 7 (60dpi) (ZIKV). Data at 1 and 30 dpi represent one of three independent experiments; 60 dpi data represent one of over two independent experiments. In b-e and g, N=10 mice/group. Data represent one of three independent experiments. In h: N=10 (mock) and 11 (ZIKV). Data represent one of two independent experiments. In i: N=19 (mock) and 22 (ZIKV). Data represent two independent experiments. In j-k: N=10 (mock) and 12 (ZIKV). Source data are provided as Source Data File.

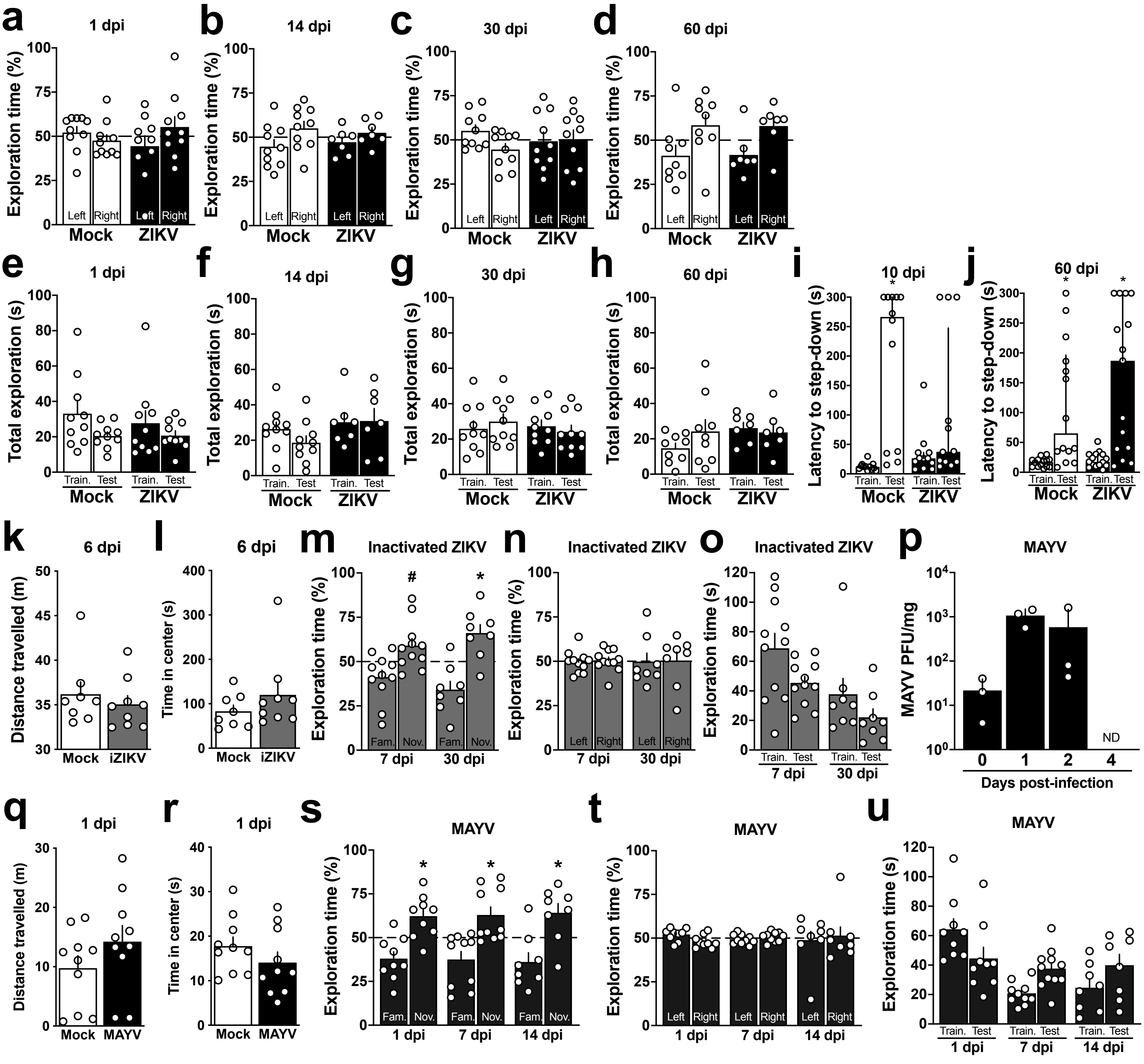

Supplementary Figure 4. ZIKV does not impair object exploration, and iZIKV and MAYV do not impair NOR memory. Mice received i.c.v. infusions of ZIKV, inactive ZIKV (iZIKV), Mayaro virus (MAYV) or mock and were tested in the NOR, open-field (30 min) or passive avoidance tests. Time exploring identical objects (left/right) during the NOR-training session (a-d, n, t), and total exploration time (s) towards both objects during NOR training or test sessions (e-h, o, u). a, c, e, g: N=10 mice/group; b, f: N=10 (mock) and 7 (ZIKV); d, h: N=9(mock) and 7 (ZIKV). n, o: N=11 (7 dpi), 8 (30 dpi). t, u: N = 9 (1 dpi), 10 (7 dpi), 8 (14 dpi). (i, j) Animals trained in the inhibitory avoidance test at 10 (i) or 60 (j) dpi. Step-down latencies. i: *p<0.0001 training vs. test for mock-infused mice, Kruskal-Wallis test followed by Dunn's; N=12 mice/group. j: *p=0.0025 training vs. test for mock-infused mice; p=0.0011 training vs. test session for ZIKV-infected mice, Kruskal-Wallis test followed by Dunn's, N=14 (mock), 15 (ZIKV). a-c, e-g and i: data represent one of two independent experiments. (k, l, q, r) iZIKV-infused or MAYV-infected mice tested in the open field (5 min) at 6 (k, l) or 1 (q, r) dpi. Total distance traveled (k, q), time at the center of the open field arena (l, r). k, l: N=8 (mock), 9 (iZIKV). q, r: N=10 mice/group. (m, s) iZIKV-infused or MAYV-infected mice did not show memory impairment in NOR. Time spent exploring novel or familiar objects during test session (m, s). (m: t=2.185, #p=0.0538 for 1 dpi; t=3.274, * p=0.0136 for 30 dpi. s: t=2.977, *p=0.0177 for 1 dpi; t=3.07, *p=0.0118 for 7 dpi; t=2.582, p=0.0364 for 14 dpi. One-sample Student's t-test compared to 50% chance level. m: N=11 (7 dpi), 8 (30 dpi). s: N=9 (1 dpi), 10 (7 dpi), 8 (14 dpi). (p) Infectious MAYV particles in mouse brain extracts determined by a plaque assay. Bars represent means ± SEM; i and j, median ± interquartile range. Symbols represent individual mice. Source data are provided as Source Data File.

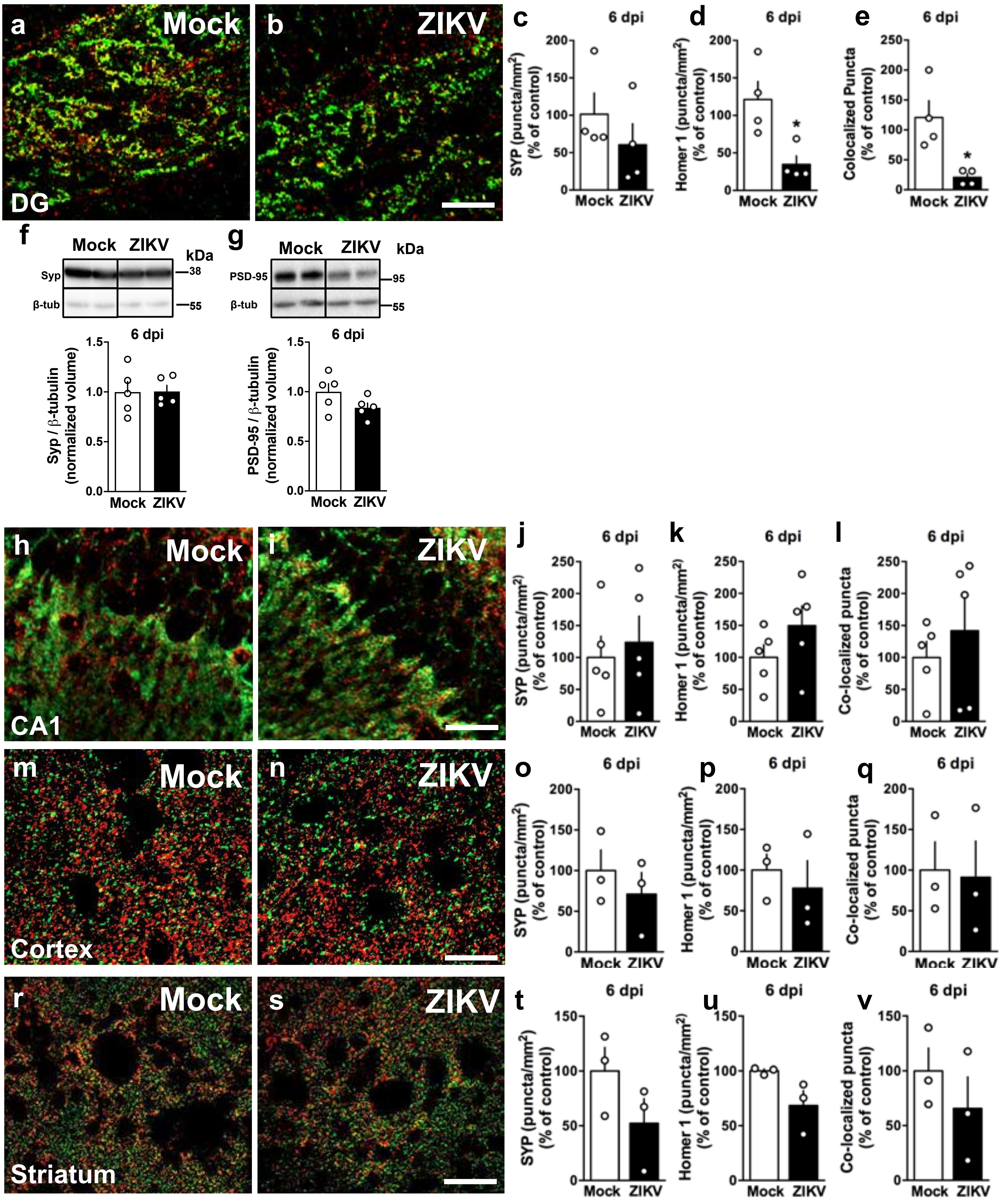

Supplementary Figure 5. ZIKV induces synapse damage in the DG, but not in CA1 hippocampal subregion, frontal cortex or striatum. Mice received an i.c.v. infusion of 105 PFU ZIKV or mock medium and brains were processed for histochemistry. Representative images of the DG (a, b) and CA1 (h, i) hippocampal regions of mock-infused (a, h) or ZIKV-infected mice (b, i) at 6dpi, immunolabeled for synaptophysin (SYP, green) and Homer1 (red). (c-e, j-l) Numbers of puncta for SYP (c, j), Homer1 (d, k) and co-localized SYP/Homer1 puncta (e, l). In c-e, j-l: N=4 mice/group. In d: t=3.258, p=0.0173; In e: t=3.484, p=0.0131). (f, g) Western blot analysis of SYP (f) or PSD-95 (g) markers in the hippocampi of mock-infused or ZIKV-infected mice at 6dpi; β-tubulin (β-tub) was used as loading control (t=2.529; p=0.0393; N=5 mice/group; representative bands (top) were from non-contiguous lanes on the same gel). (m, n) Representative images of the parietal cortex of mock-infused (m) or ZIKV-infected mice (n) at 6dpi, immunolabeled for SYP (green) and Homer1 (red). (o-q) Numbers of puncta for SYP (o), Homer1 (p) and co-localized SYP/Homer1 puncta (q) in the parietal cortex. N=3 mice/group. (r, s) Representative images of the striatum of mock-infused (r) or ZIKV-infected mice (s) at 6dpi, immunolabeled for SYP (green) and Homer1 (red). (t-v) Number of puncta for SYP (t), Homer1 (u) and co-localized SYP/Homer1 puncta (v) in the striatum. N=3 mice/group. Scale bar = 25 µm. Source data from panels c-g, j-l, o-q, t-v are provided as Source Data File.

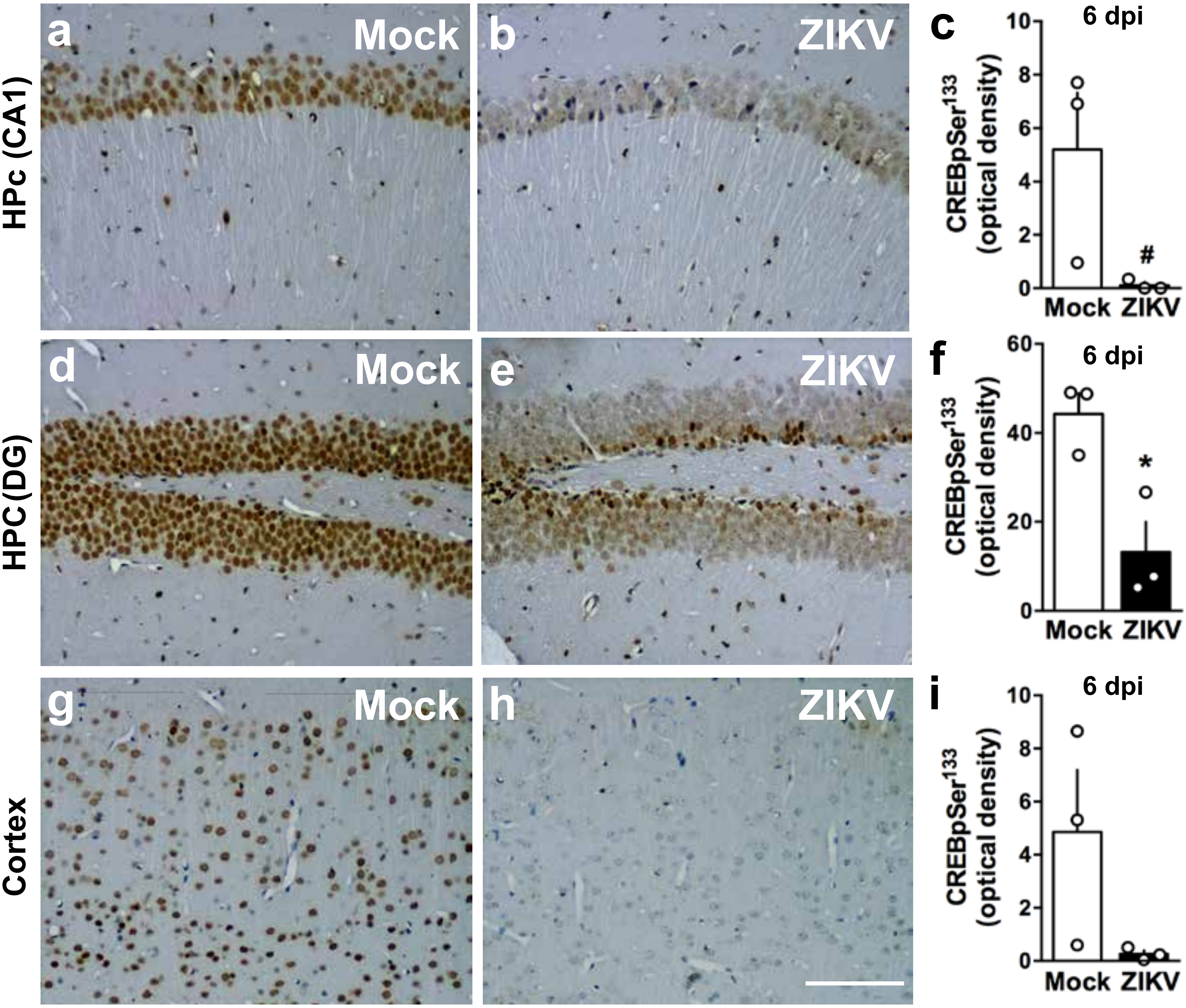

Supplementary Figure 6. ZIKV infection in adult mice leads to decreased CREB phosphorylation. Mice received an i.c.v. infusion of 10^5 PFU ZIKV or mock medium, and brains were processed for histochemistry. (a, b, d, e, g, h) Representative images showing immunolabeling for cAMP-responsive element binding protein (CREB) phosphorylated at serine 133 (CREBpSer^{133}) in hippocampal CA1 (a, b), dentate gyrus (DG) (d, e) and in the parietal cortex (g, h) of mock- or ZIKV-infected mice at 6dpi. Scale bar=50 µm (c, f, i) Graphs show integrated CREBpSer^{133} immunoreactivities (optical density) in the indicated regions (In c; t=2.381; #p=0.0759; In f; t=3.787; *p=0.0193; Student's t-test; N=3 mice/group). Bars represent means ± SEM. Symbols correspond to individual mice. Source data from panels c, f, i are provided as Source Data File.

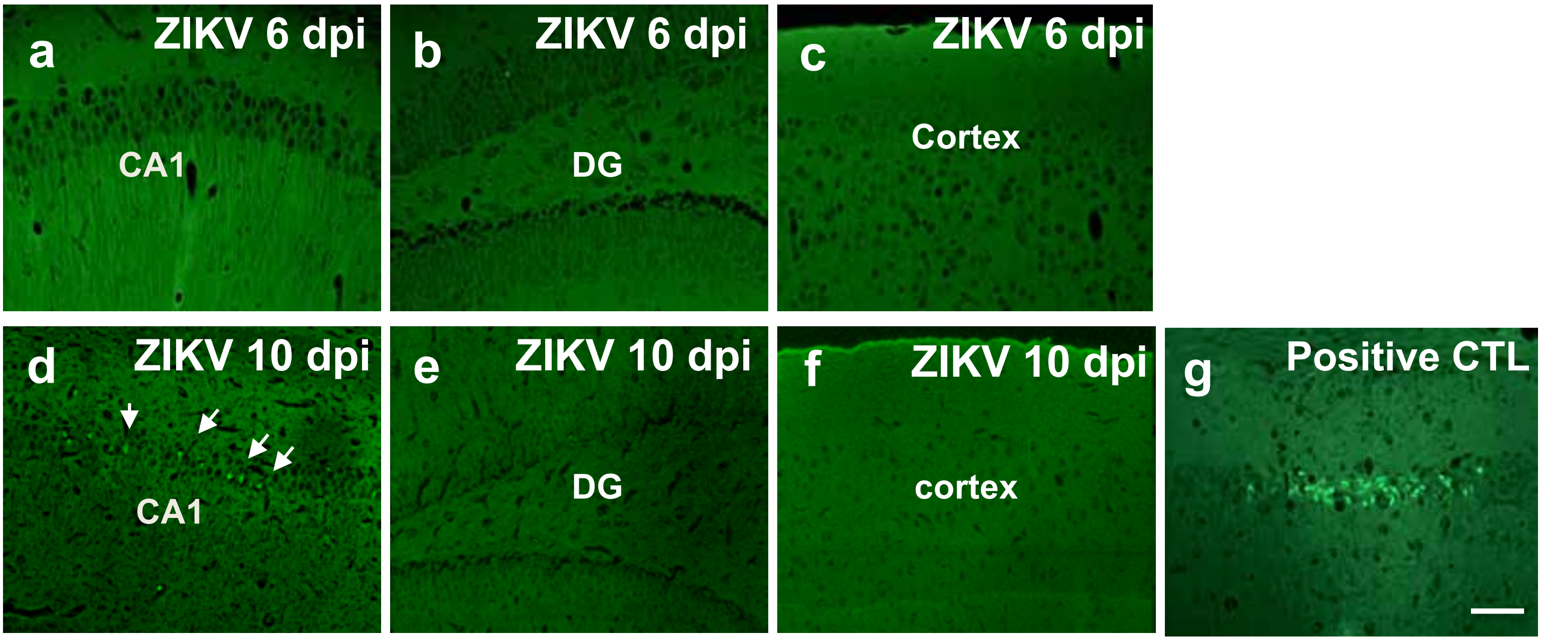

Supplementary Figure 7. ZIKV does not induce neuronal death in the hippocampus of adult mice. Mice received an i.c.v. infusion of 10^5 PFU ZIKV or mock medium, and brains were processed for histochemistry. (a-f) Representative Fluorojade B staining in the CA1 (a, d), DG (b, e) and parietal cortex (c, f) of ZIKV-infused mice at 6 (a-c) or 10 dpi (d-f). N=6 mice/group (6 dpi) and 5 mice/group (10 dpi) (g) FluoroJade B staining positive control consisted of brain sections of a mouse infused i.c.v. with the neurotoxin quinolinic acid. Scale bar = 50 μm.

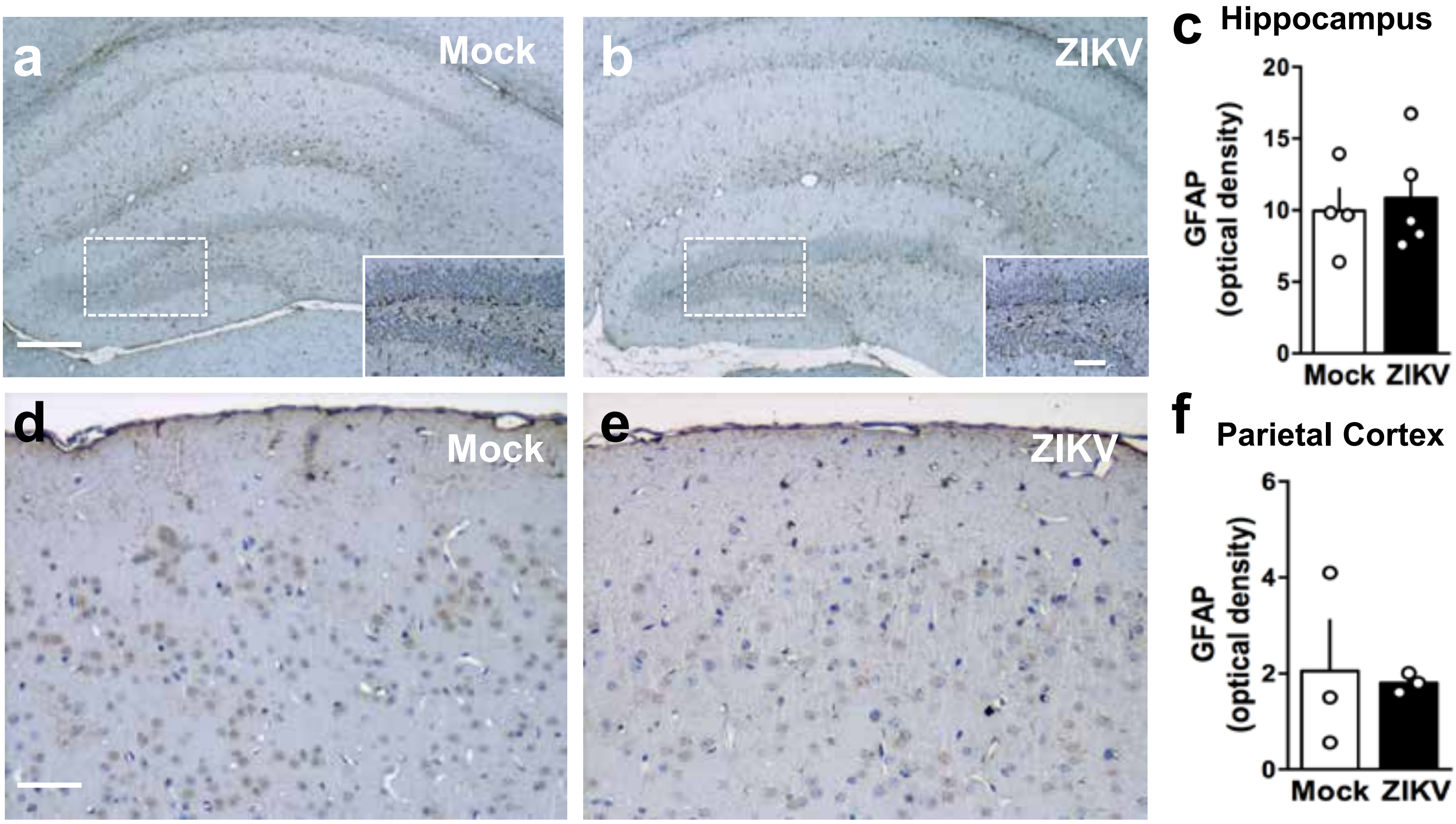

Supplementary Figure 8. ZIKV does not induce astrogliosis in adult mice. Mice received an i.c.v. infusion of 10^5 PFU ZIKV or mock medium, and brains were processed for histochemistry. Representative images of GFAP immunolabeling in the hippocampus (a, b) and parietal cortex (d, e) of mock- or ZIKV-infused mice at 6dpi. Scale bars = 200 μm. Insets show higher magnification images of the regions defined by dashed white rectangles in panels. Scale bar insets = 50 μm. (c, f) Integrated GFAP immunoreactivities (optical density) from images acquired from the hippocampus (c) and parietal cortex (f) of mock- and ZIKV-infused mice at 6 dpi. In c: N=4 (mock) and 5 (ZIKV); in f: N=3 mice/group. Bars represent means ± SEM. Symbols correspond to individual mice. Source data from panels c and f are provided as Source Data File.

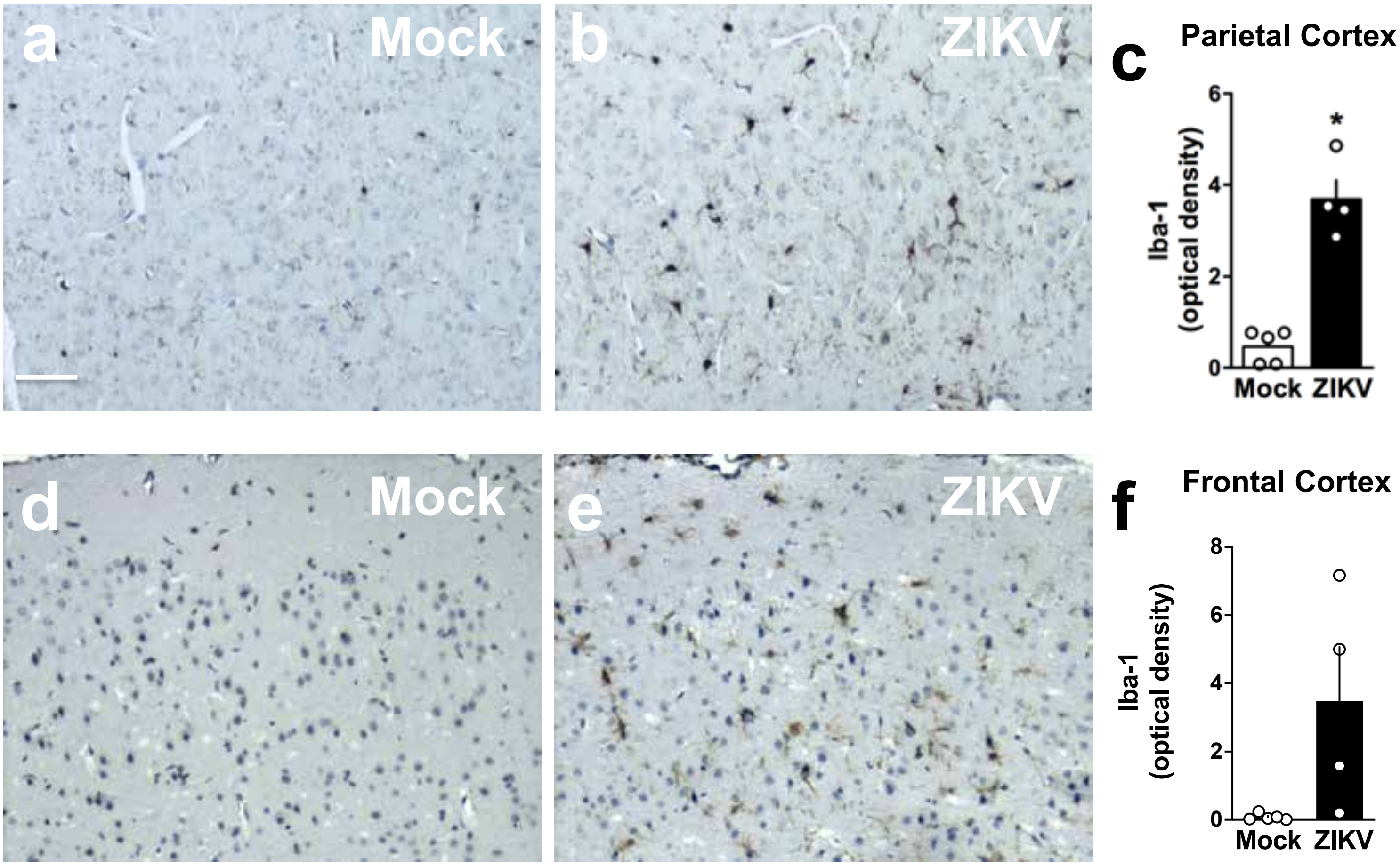

Supplementary Figure 9. ZIKV induces microgliosis in the parietal and frontal cortices of adult mice. Mice received an i.c.v. infusion of 10^5 PFU ZIKV or mock medium, and brains were processed for histochemistry. Representative images of Iba-1 immunohistochemistry in the parietal (a, b) and frontal (d, e) cortices from mock- or ZIKV-infused mice at 10 dpi. Scale bar=200 μm. (c, f) Integrated Iba-1 immunoreactivities (optical density) from images acquired from the parietal (c) or frontal (f) cortices. (t=7.814, *p=0.0001, Student's t-test). N=5 (mock) and 4 (ZIKV) mice. Bars represent means ± SEM. Symbols correspond to individual mice. Source data from panels c and f are provided as Source Data File.

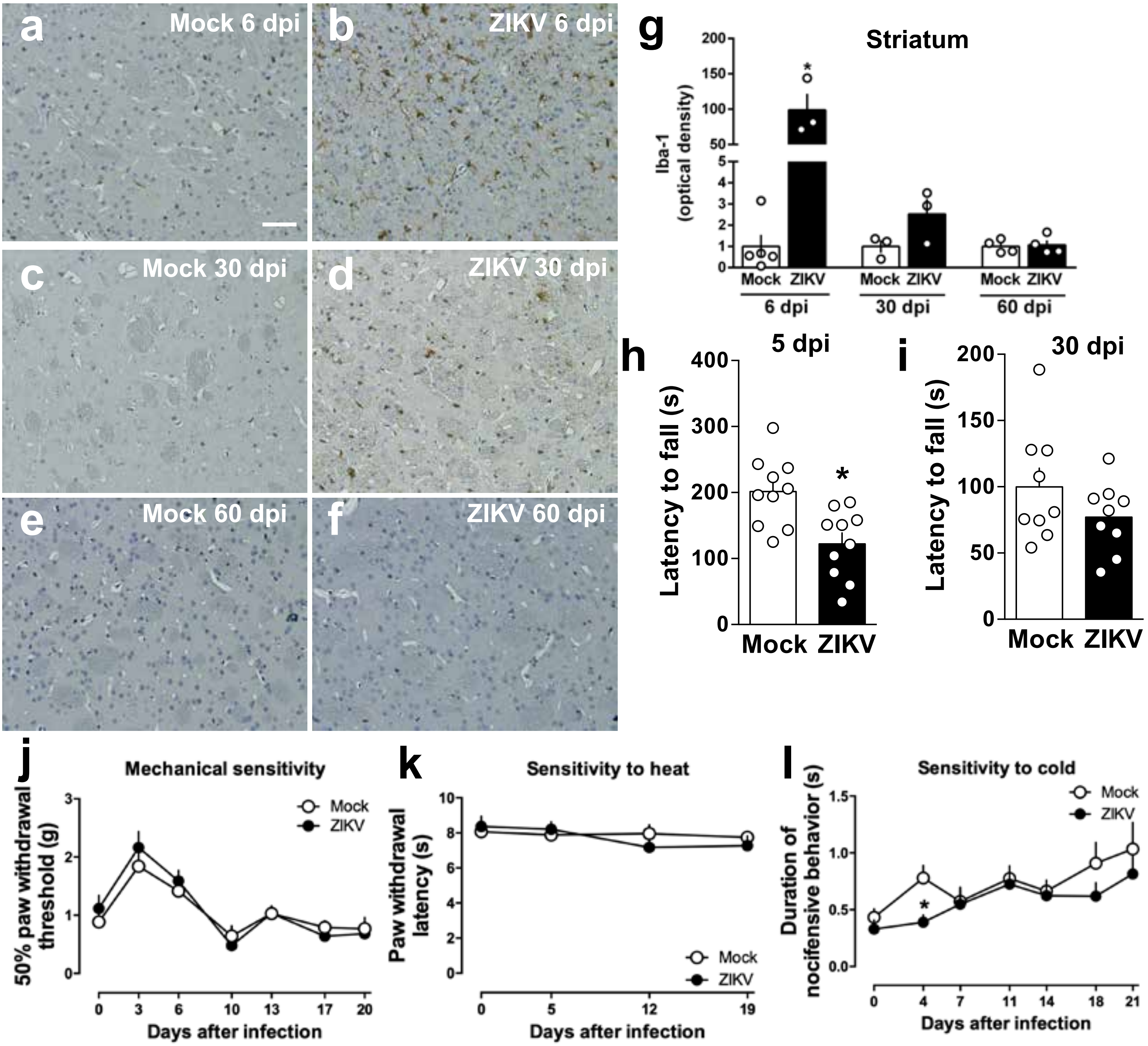

Supplementary Figure 10. ZIKV induces striatal microgliosis and causes motor, but not sensory, deficits in adult mice. Mice received an i.c.v. infusion of 10^5 PFU ZIKV or mock medium, and brains were processed for histochemistry. (a-f) Representative images of Iba-1 immunoreactivity in the striatum of mock- or ZIKV-infused mice at 6 (a, b), 30 (c, d) or 60 (e, f) dpi. Scale bar=200 μm. (g) Integrated Iba-1 immunoreactivities (optical density) from images acquired from the striatum of mice at different dpi. ($F(2,16)=29.73$, 6 dpi: *$p<0.0001$; two-way ANOVA followed by Bonferroni compared to mock at the same time-point; N=5 (6 dpi), 3 (30 dpi) and 4 (60 dpi) (mock) and 3 (6 dpi), 3 (30 dpi) and 4 (60 dpi) (ZIKV). (h, i) Animals were tested in the Rotarod task at 5 (h) or 30 (i) dpi (in h: $t=3.391$, *$p=0.0033$.; N=10 mice/group; data represent one of three independent experiments; in i: N=10 mice/group). (j-l) ZIKV or mock-infused animals were tested for mechanical (j), heat (k) or cold (l) sensitivities at indicated time points. Bars represent means ± SEM. Symbols correspond to individual mice. (N=10 mice/group). Source data from panels g-l are provided as Source Data File.

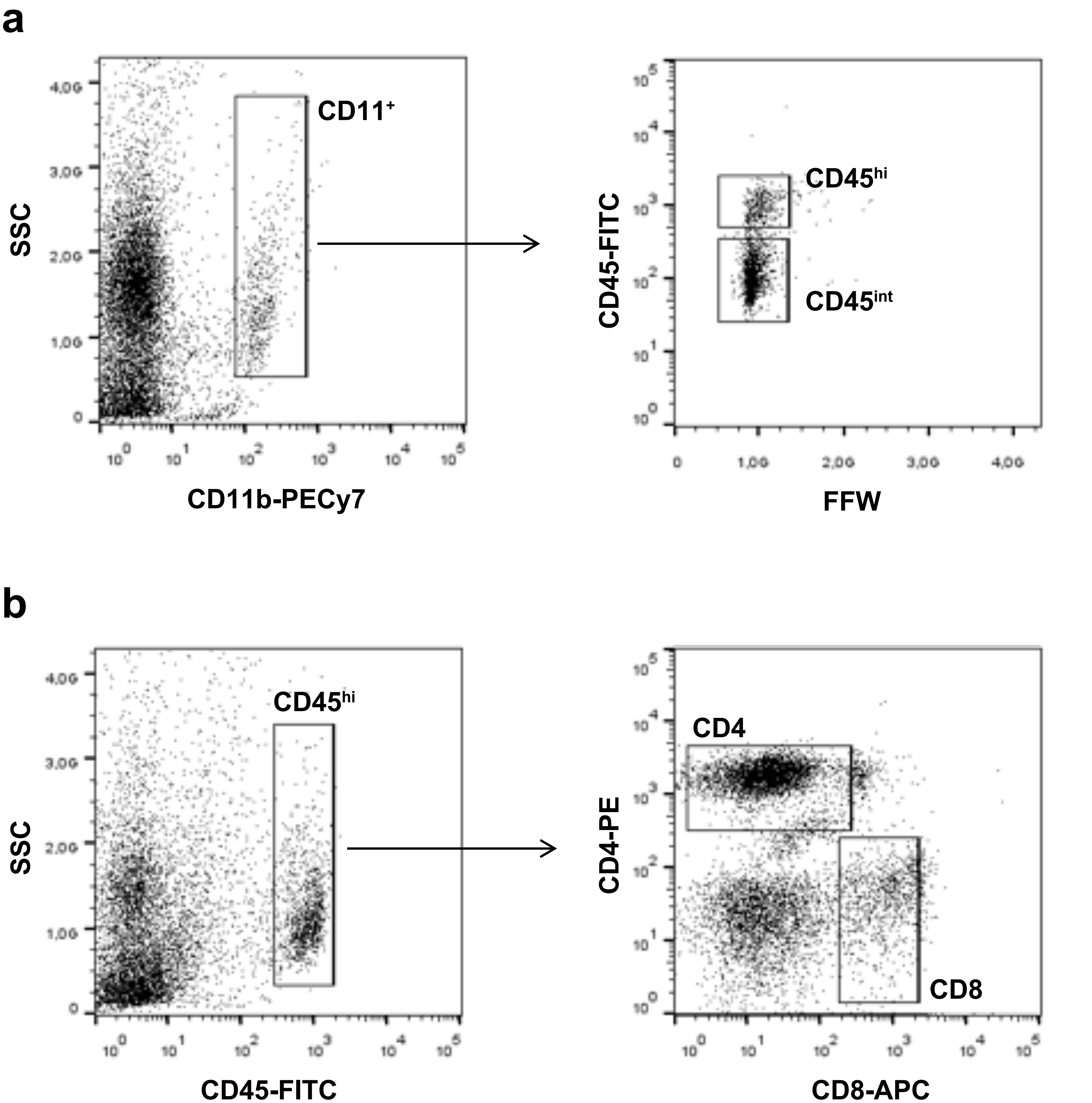

Supplementary Figure 11. Gating strategies used for analysis of brain infiltrating and resident leukocytes. (a) Gating strategy to analyze brain resident (CD11b+ CD45int) and infiltrating myeloid cells as presented in Figure 4p-r. (b) Gating strategy to analyze brain CD4 (CD45hi CD4+) and CD8 (CD45hi CD8+) T lymphocytes as presented in Figure 4s-u.

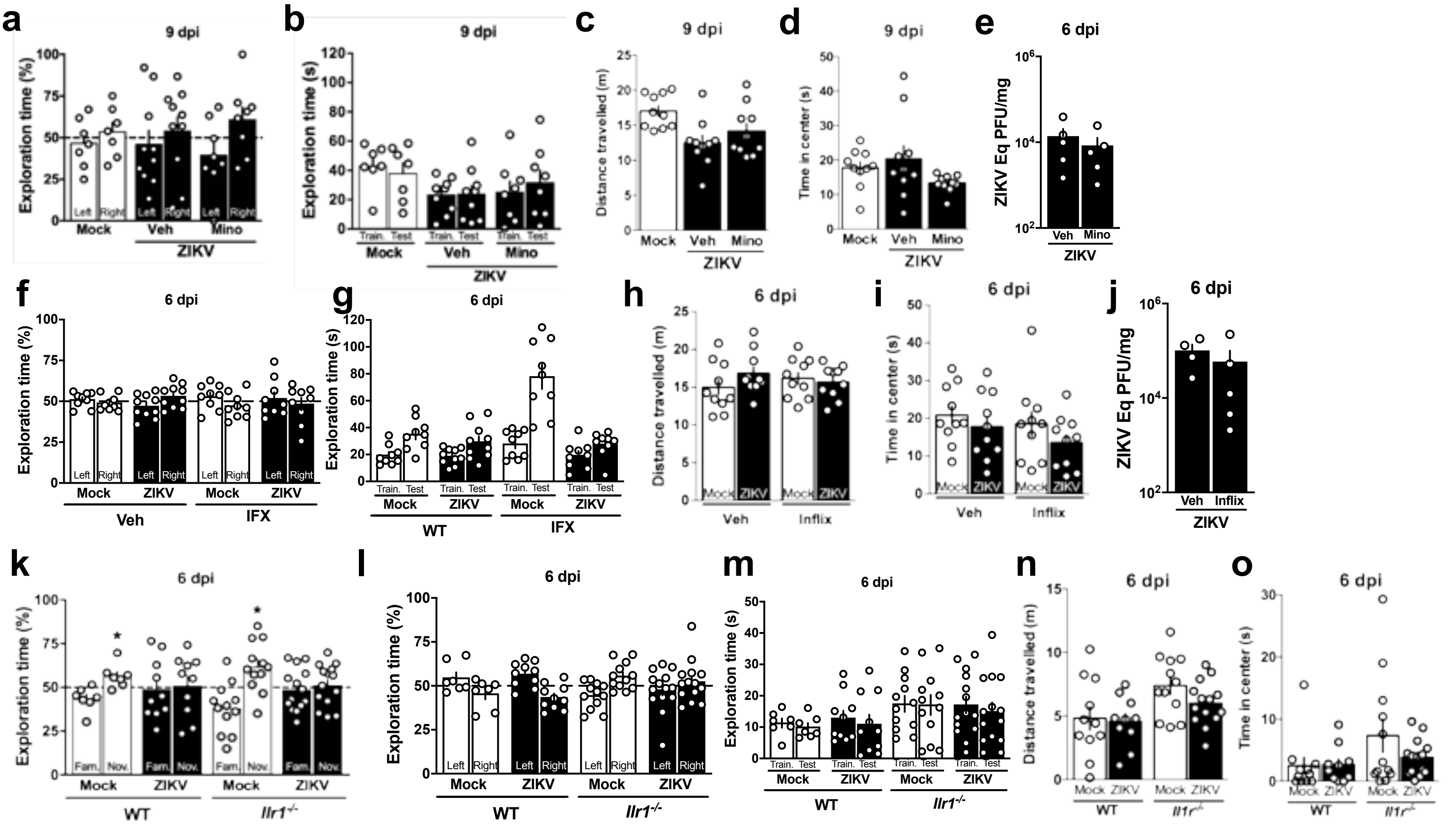

Supplementary Figure 12. Minocycline or infliximab had no effects on locomotor/exploratory activities in an open field, or anxiety behavior in adult mice. Mice received an i.c.v. infusion of 10^5 PFU ZIKV or mock medium and were then treated with minocycline (Mino) (a-e) or infliximab (Inflix) (f-i) (e, j) ZIKV mRNA levels in the hippocampi of ZIKV-infected mice treated with minocycline (e) or infliximab (j) at 6 dpi. (k-o) Adult wild type (WT) (C57BL/6) or $Il1r^{-/-}$ mice received an i.c.v. infusion of 105 PFU ZIKV or mock medium. Animals were tested in the open field and novel object recognition (NOR) tests at indicated dpi. Graphs show total distance traveled (c, h, n), time spent at the center of the open field arena (d, i, o), % time spent exploring identical objects during the training session (a, f, l), and total time exploring both objects during training (train.) or test sessions (b, g, l) of NOR. (k) Time spent exploring the novel (Nov) or familiar (Fam) objects during test session (*t=2.553 p=0.043 for Mock/WT; t=2.897 p=0.0145 for Mock/$Il1r^{-/-}$; one-sample Student's t-test compared to the chance value of 50%). In a, b: N=8 (ZIKV+mino), 7 (mock) and 10 (ZIKV+Veh); in c, d: N=10 mice/group; in e: N = 5 mice/group. in f, g: N=10 (ZiKV) and 9 (other groups). in h, i: N=10 mice/group; in j: N = 4 (ZIKV) and 5 (ZIKV+Inflix) mice; in k, l, m: N=7 (Mock/WT), 10 (ZIKV/WT), 12 (mock/$Il1r^{-/-}$) and 14 (ZIKV/$Il1r^{-/-}$) mice; in n, o: 11 (Mock/WT), 10 (ZIKV/WT), 12 (Mock/$Il1r^{-/-}$) and 14 (ZIKV/$Il1r^{-/-}$) mice. Bars represent means ± SEM. Symbols correspond to individual mice. In a-d and f-l data represent one of two independent experiments. Source data are provided as Source Data File.

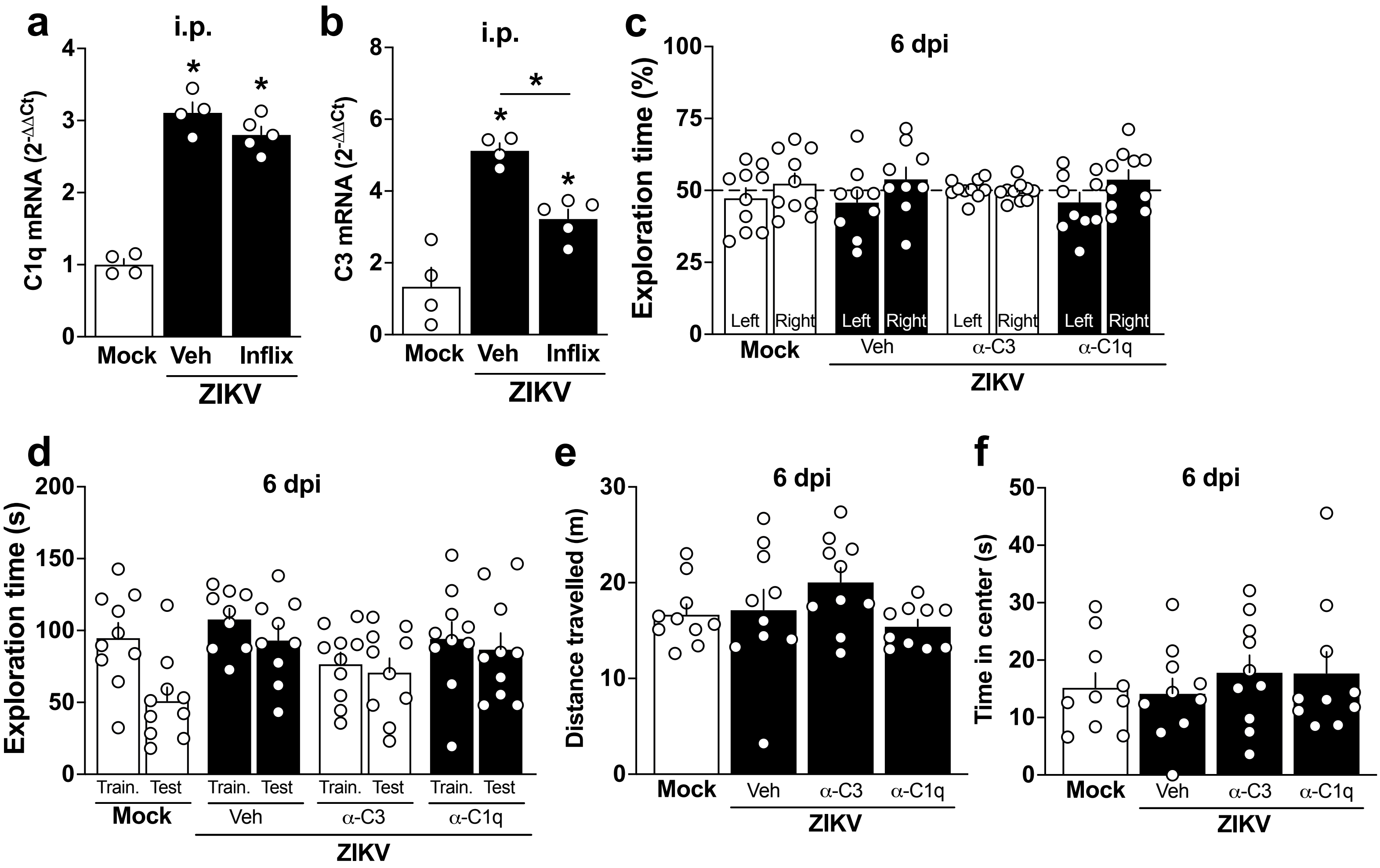

Supplementary Figure 13. Neutralization of brain complement system proteins does not affect locomotor/exploratory activities in an open field or anxiety behavior in adult mice. (a, b) Mice were treated with infliximab (Inflix) i.p. and received an i.c.v. infusion of ZIKV (10^5 PFU) or mock medium. At 6 dpi, brains were processed for determination of expression of C1q (a: $F(2,10)=99.0$, *$p<0.001$; one-way ANOVA followed by Bonferroni; and C3 (b: $F(2,10)=29.0$, *$p<0.0001$ for Mock vs. ZIKV, $p=0.0075$ for Mock vs. ZIKV+Inflix, $p=0.0072$ for ZIKV vs. ZIKV+Inflix; one-way ANOVA followed by Bonferroni; N=4 (mock and ZIKV+veh) and 5 (ZIKV+Inflix) mice). (c-f) Mice received an i.c.v. infusion of ZIKV (105 PFU) or mock medium and were treated with antibodies against C3 or C1q i.c.v. at 4 and 5 dpi. Mice were tested in the open-field and novel object recognition (NOR) tests at 6 dpi. Graphs show time spent exploring identical objects (left and right) during the training session (c), total time exploring both objects during training (train.) and test sessions (d) of NOR, total distance traveled in the open-field arena (e) and time spent at the center of the open field arena (f). In c, d: N=9 (ZIKV+Veh) and 10 (each other group) mice; in e, f: N=10 mice/group. Bars represent means±SEM. Symbols correspond to individual mice. Source data are provided as Source Data File.
