# Supplementary material for: Zika virus replicates in adult human brain tissue and impairs synapses and memory in mice
Source: Nat Commun. 2019 Sep 5;10:3890. doi: 10.1038/s41467-019-11866-7 (PMC6728367; doi:10.1038/s41467-019-11866-7)
Supplement: Supplementary file 3 — Description of Additional Supplementary Files [file 41467_2019_11866_MOESM3_ESM.docx]

**Description of Additional Supplementary Files**

**File Name: Supplementary Movie 1**

**Description:** Synaptic engulfment by microglia in the hippocampus of ZIKV-infected mice. 3D-median reconstructions from Z-stack images acquired in the CA3 hippocampal region from ZIKV-infected mice, showing synaptic marker 4 synaptophysin (green puncta) profiles inside microglia (Iba-1, red).

**File Name: Supplementary Movie 2**

**Description:** Synaptic engulfment by microglia in the hippocampus of mock-infused mice. 3D-median reconstructions from Z-stack images acquired in the CA3 hippocampal region from mockinfused mice, showing synaptic marker synaptophysin (green puncta) and microglia (Iba-1, red).
