# Supplementary material for: Zika virus replicates in adult human brain tissue and impairs synapses and memory in mice
Source: Nat Commun. 2019 Sep 5;10:3890. doi: 10.1038/s41467-019-11866-7 (PMC6728367; doi:10.1038/s41467-019-11866-7)
Supplement: Supplementary file 6 — Reporting Summary [file 41467_2019_11866_MOESM6_ESM.pdf]

nature research

Corresponding author(s): Sérgio T. Ferreira
Claudia P. Figueiredo
Andrea T. Da Poian

Last updated by author(s): Jul 11, 2019

Reporting Summary

Nature Research wishes to improve the reproducibility of the work that we publish. This form provides structure for consistency and transparency in reporting. For further information on Nature Research policies, see Authors & Referees and the Editorial Policy Checklist.

Statistics

For all statistical analyses, confirm that the following items are present in the figure legend, table legend, main text, or Methods section.

n/a	Confirmed	
☐	☒	The exact sample size (*n*) for each experimental group/condition, given as a discrete number and unit of measurement
☐	☒	A statement on whether measurements were taken from distinct samples or whether the same sample was measured repeatedly
☐	☒	The statistical test(s) used AND whether they are one- or two-sided<br>*Only common tests should be described solely by name; describe more complex techniques in the Methods section.*
☒	☐	A description of all covariates tested
☐	☒	A description of any assumptions or corrections, such as tests of normality and adjustment for multiple comparisons
☐	☒	A full description of the statistical parameters including central tendency (e.g. means) or other basic estimates (e.g. regression coefficient) AND variation (e.g. standard deviation) or associated estimates of uncertainty (e.g. confidence intervals)
☐	☒	For null hypothesis testing, the test statistic (e.g. *F*, *t*, *r*) with confidence intervals, effect sizes, degrees of freedom and *P* value noted<br>*Give P values as exact values whenever suitable.*
☒	☐	For Bayesian analysis, information on the choice of priors and Markov chain Monte Carlo settings
☒	☐	For hierarchical and complex designs, identification of the appropriate level for tests and full reporting of outcomes
☒	☐	Estimates of effect sizes (e.g. Cohen's *d*, Pearson's *r*), indicating how they were calculated

Our web collection on statistics for biologists contains articles on many of the points above.

Software and code

Policy information about availability of computer code

Data collection	Image Lab Software v. 6.0.1 Bio-Rad. Applied Biosystems 7500 RT–PCR system. Axoclamp 2A (Axon Instruments, USA). MoFlo XDP FACS equipment (Beckman Coulter Inc., Brea, CA).
Data analysis	ANY-maze behavioral tracking software v. 4.99 Stoelting Co. Image Lab Software v. 6.0.1 Bio-Rad. Applied Biosystems 7500 RT–PCR system. ImageJ 1.29 (NIH; RRID: SCR_003070). FIJI + Puncta Analyzer (v2.0 plugin, NIH Image J). Axoclamp 2A (Axon Instruments, USA). FlowJo vX software (Tree Star Inc., Ashland, OR). GraphPad Prism v.8.1.2

For manuscripts utilizing custom algorithms or software that are central to the research but not yet described in published literature, software must be made available to editors/reviewers. We strongly encourage code deposition in a community repository (e.g. GitHub). See the Nature Research guidelines for submitting code & software for further information.

Data

Policy information about availability of data

All manuscripts must include a data availability statement. This statement should provide the following information, where applicable:
- Accession codes, unique identifiers, or web links for publicly available datasets
- A list of figures that have associated raw data
- A description of any restrictions on data availability

Data underlying Fig. 1b; Fig. 2b-e; Fig. 3a-f, i-k, n-t; Fig. 4a-e,o, q, r, t, u; Fig. 5c, g-n; Fig. 6d-e, i-p; Fig. 7 a-f, k-n, Suppl. Fig. 1; Suppl. 2f; Suppl. Fig. 3; Suppl. Fig. 4; Suppl. Fig. 5 c-g, j-l, o-q, t-v; Suppl. Fig. 6c, f, i; Suppl. Fig. 8c, f; Suppl. Fig. 9c, f; Suppl. Fig. 10 g-l; Suppl. Fig. 12; Suppl. Fig. 13 are provided as Source Data files. All other data are available from the corresponding authors upon reasonable requests.

Field-specific reporting

Please select the one below that is the best fit for your research. If you are not sure, read the appropriate sections before making your selection.

☒ Life sciences ☐ Behavioural & social sciences ☐ Ecological, evolutionary & environmental sciences

For a reference copy of the document with all sections, see nature.com/documents/nr-reporting-summary-flat.pdf

Life sciences study design

All studies must disclose on these points even when the disclosure is negative.

Sample size	Sample size for each experiment was calculated based upon pilote studies and the formula n = (Saˆ2+Sbˆ2)x([Za/2+Zb/2)/d]ˆ2. Sa and Sb are standard deviations for each experimental group. Za/2 = 1.96 (5%) and Zb = 0.84 (20%). d is the minimum difference from media.
Data exclusions	Mice showing any signs of misplaced intracerebroventricular injections or brain hemorrhage (~5% of animals throughout our study) were excluded from further analysis. In novel object experiments, mice that did not explore one or both objects were excluded.
Replication	All attempts of replication were successful.
Randomization	Mice were radomly assigned to experimental groups.
Blinding	Experimenters were blind to experimental conditions during animal behavioral test analysis and synaptic plasticity analysis.

Reporting for specific materials, systems and methods

We require information from authors about some types of materials, experimental systems and methods used in many studies. Here, indicate whether each material, system or method listed is relevant to your study. If you are not sure if a list item applies to your research, read the appropriate section before selecting a response.

Materials & experimental systems

n/a	Involved in the study	
☐	☒	Antibodies
☒	☐	Eukaryotic cell lines
☒	☐	Palaeontology
☐	☒	Animals and other organisms
☐	☒	Human research participants
☒	☐	Clinical data

Methods

n/a	Involved in the study	
☒	☐	ChIP-seq
☐	☒	Flow cytometry
☒	☐	MRI-based neuroimaging

Antibodies

Antibodies used	rabbit anti-GFAP, 1:500, DAKO Z0334; rabbit anti-Iba-1, 1:1,000, Wako NCNP24; rabbit anti- CREB pSer133, 1:100, Cell Signaling #9198; rabbit anti-NS2B, 1:50, GeneTex #133308. rabbit anti-NS2B, 1:50, GeneTex #133308; mouse anti-GFAP, 1:500, Sigma #G3893; rat anti-F4/80, 1:25, Bio-Rad #MCA497; mouse anti-NeuN, 1:50, Chemicon #MAB377; rabbit monoclonal anti-TMEM119, 1:50, Abcam #210405; mouse anti-DCX 1:50, Santa Cruz #271390, mouse anti-synaptophysin 1:200, Vector Laboratories #S285; rabbit anti-Homer-1 1:100, Abcam #184955. Alexa 555-, 594- or 488-conjugated secondary antibodies (1:750; Invitrogen).
Validation	Antibodies were validated using positive and negative controls for staining.

Animals and other organisms

Policy information about studies involving animals; ARRIVE guidelines recommended for reporting animal research

Laboratory animals	Male Swiss mice, male C57BL/6 mice or male Il1r-/-mice from our animal facility were 2.5-3 month-old at the beginning of experiments. A129 mice were 5-8 weeks-old at the beginning of experiments. Neonatal (post-natal day 3) Swiss-mice.
Wild animals	N/A.
Field-collected samples	N/A.
Ethics oversight	All procedures followed the "Principles of Laboratory Animal Care" (US National Institutes of Health) and were approved by the Institutional Animal Care and Use Committee of the Federal University of Rio de Janeiro (protocols #043/2016 and #126/2018).

Note that full information on the approval of the study protocol must also be provided in the manuscript.

Human research participants

Policy information about studies involving human research participants

Population characteristics	Samples of temporal lobe cortical tissue were obtained from patients with drug-resistant epilepsy subjected to temporal lobectomy.
Recruitment	Patients with drug-resistant epilepsy that would be subjected to temporal lobectomy in our university hospital (Hospital Universitário Clementino Fraga Filho) entered the study.
Ethics oversight	All procedures were approved by the National Committee for Research Ethics of the Brazilian Ministry of Health (protocol #0069.0.197.000-05). Informed consent was obtained from all participants.

Note that full information on the approval of the study protocol must also be provided in the manuscript.

Flow Cytometry

Plots

Confirm that:

☒ The axis labels state the marker and fluorochrome used (e.g. CD4-FITC).

☒ The axis scales are clearly visible. Include numbers along axes only for bottom left plot of group (a 'group' is an analysis of identical markers).

☒ All plots are contour plots with outliers or pseudocolor plots.

☒ A numerical value for number of cells or percentage (with statistics) is provided.

Methodology

Sample preparation	Sample preparation is described in manuscript Methods session.
Instrument	MoFlo XDP FACS equipment (Beckman Coulter Inc., Brea, CA).
Software	FlowJo vX software (Tree Star Inc., Ashland, OR).
Cell population abundance	N/A
Gating strategy	Described in Suppl. Fig. 11.

☒ Tick this box to confirm that a figure exemplifying the gating strategy is provided in the Supplementary Information.
